# Weight Loss After Obesity Disrupts Cognitive Flexibility Through Reinforcement Learning Strategies

**DOI:** 10.1002/oby.70057

**Published:** 2025-10-28

**Authors:** Yufan Li, Reema Sharma, Abigail Usiyevich, Xiwen Shen, Kelly W. Zhang, Koulik Khamaru, Bridget A. Matikainen‐Ankney

**Affiliations:** ^1^ Behavioral and Systems Neuroscience, Psychology Department Rutgers University Piscataway New Jersey USA; ^2^ Bank of Suzhou Co Suzhou Jiangsu China; ^3^ Department of Mathematics Imperial College London London UK; ^4^ Department of Statistics Rutgers University Piscataway New Jersey USA

## Abstract

**Objective:**

Despite successful weight loss, many individuals with obesity regain weight, yet cognitive factors in the weight loss state remain unclear. Here, we tested whether obesity induces deficits in cognitive flexibility, a core component of reinforcement learning (RL), after body weight normalizes.

**Methods:**

Male and female C57BL/6J mice were exposed to high‐fat diet‐induced obesity followed by weight loss. Weight loss and control mice were tested on a modified probabilistic reversal learning (PRL) task to assess cognitive flexibility and a progressive‐ratio (PR) task to evaluate motivation. RL modeling was applied to dissociate latent decision‐making parameters.

**Results:**

Post‐weight‐loss mice exhibited persistent impairments in PRL efficiency. Males showed reduced late‐phase reversal efficiency (*p* < 0.001), while females showed early‐phase inefficiency but later recovery (*p* < 0.05). RL modeling revealed reduced learning rates in both sexes, indicating impaired value updating despite intact motivation, as PR performance did not differ between groups. Across tasks, food intake remained unchanged, suggesting reduced efficiency reflected cognitive inflexibility rather than diminished appetite.

**Conclusions:**

Weight loss after obesity produced sex‐specific RL deficits. These findings dissociated motivational drive from cognitive flexibility and highlighted maladaptive decision‐making as a feature of the weight loss state. This demonstrates the need for targeted interventions addressing post‐weight‐loss cognitive barriers.

**Study Importance**

- What is already known?
  ○ Cognitive flexibility—the ability to adapt behavior in response to changing environmental contingencies—is reduced in individuals with obesity.
  ○ Deficits in cognitive flexibility have been implicated in compulsive behaviors and relapse in addiction‐like models, but little is known about whether these deficits persist after weight loss.
- What does this study add?
  ○ Even after successful weight loss and normalized body weight, previously obese mice exhibited lasting impairments in cognitive flexibility in the weight loss state, as shown by reduced performance on probabilistic reversal learning tasks.
  ○ Males exhibited late‐phase inefficiencies during reversal learning, suggesting persistent inflexibility, whereas females showed early‐phase deficits but were able to adjust dynamically and recover later.
- How might these results change the direction of research or the focus of clinical practice?
  ○ Targeting cognitive flexibility and reinforcement learning deficits after weight loss may be a crucial—but currently overlooked—strategy to prevent weight regain.
  ○ The observed sex differences suggest that men and women may benefit from distinct cognitive‐behavioral or pharmacological approaches. Female‐tailored interventions may focus on supporting early adaptation, while male interventions may need to address persistent rigidity.

## 1 Introduction

With the growing availability of easily accessible palatable foods, diet‐induced overweight and obesity have become global health challenges, especially in the United States, with a 40.3% prevalence in adults [1]. Efforts to promote weight loss through medical, surgical, and pharmacological interventions reduce the risk of obesity‐related diseases [2, 3, 4]. However, even after successful weight loss, the majority of individuals often suffer from weight regain, counteracting metabolic benefits from weight loss and perpetuating physical and mental health burdens throughout the course of the weight loss and regain period [5]. This relapse highlights critical gaps in our understanding of post‐weight‐loss effects on behavior. While research has prioritized strategies to achieve weight loss, often via pharmacological treatments, the mechanisms underlying sustained weight loss maintenance—particularly cognitive and motivational barriers to sustained weight maintenance—remain poorly understood.

Growing evidence suggests that obesity disrupts food reward processing—a complex interplay of homeostatic and non‐homeostatic mechanisms. Homeostatic regulation balances energy intake with metabolic needs, while non‐homeostatic drivers—such as the hedonic properties of food, learned associations, and past experiences—prioritize reward over satiety [6, 7, 8, 9]. Palatable, calorie‐dense foods can hijack behavioral responses to food cues [10]. Over time, this dysfunction creates a self‐reinforcing cycle: increasing hedonic motivation for hyperpalatable foods overrides homeostatic needs, driving compulsive consumption resistant to satiety feedback [10]. Critically, whether these disruptions persist after weight loss—and how they interact with adaptive decision‐making—remains unknown.

Cognitive flexibility, the ability to update behaviors in response to changing rewards, is central to resisting relapse. This executive function enables individuals to abandon maladaptive habits (e.g., preference for hyperpalatable foods) and adopt new strategies aligned with metabolic goals [11, 12]. Obesity is associated with reduced cognitive flexibility in people, as evidenced by impaired reversal learning and perseverative errors in decision‐making tasks [13, 14, 15]. Compromised flexibility may explain why individuals struggle to maintain behavioral changes after weight loss: they become “stuck” in rigid patterns, unable to adapt to shifting environmental cues (e.g., food availability) [13, 14, 15]. However, cognitive flexibility in the post‐weight‐loss phase—particularly its interaction with food motivation—is poorly characterized.

To address these gaps, we developed a mouse model of post‐obesity weight loss (wgl) to investigate interactions between food motivation and cognitive flexibility. Using a modified probabilistic reversal learning (PRL) task with 80:20 reward contingencies, we modeled real‐world decision‐making under uncertainty, where high‐probability choices are inconsistently rewarded. This design dissociates reward integration from deterministic feedback, mimicking the ambiguous reinforcement inherent to natural environments [16]. We also paired it with progressive‐ratio (PR) tasks to quantify effort allocation for hedonic (sucrose) vs. homeostatic (stand grain) rewards.

To uncover the latent cognitive mechanisms underlying these behaviors, we applied a reinforcement learning (RL) model to trial‐by‐trial choice data based on Rescorla–Wagner learning theory, which explains a choice as a result of changes in prediction error across learning [17]. Traditional behavioral metrics (e.g., accuracy, poke‐per‐pellet ratio) provide insight into overall performance, while the RL model quantifies core parameters such as learning rate (speed of value updating) and inverse temperature (choice stochasticity), revealing how post‐weight‐loss mice integrate reward history and adjust strategies dynamically.

## 2 Methods

### 2.1 Animals

Fourteen male and fourteen female C57BL/6J mice were individually housed in a 12 h light/dark cycle (7 a.m. to 7 p.m. light cycle) with ad libitum access to food and water unless otherwise noted. Mice were provided with lab chow (5001 Rodent Diet) or 60% high‐fat diet (HFD) (Research Diets, D12492). Mice were weighed prior to being distributed across control or wgl groups, to ensure balanced initial weights. During 8 weeks of weight gain and 6 weeks of weight loss, mice were weighed weekly. All animals were included for all experiments and analyses, and all data generated were included for all analyses presented here. A sample size of seven mice per sex per group was calculated to ensure power > 0.8 [18, 19]. Mice were distributed to control or wgl groups to ensure that group weights were equal prior to the start of HFD feeding. All procedures were approved by the Rutgers University Animal Care and Use Committee.

### 2.2 Home Cage Operant Behavior

All behavioral tests were performed immediately after mice had lost weight for 6 weeks. Operant behavior was tested using the Feeding Experimentation Device 3 (FED3, [20]). A nose poke on the active port yielded a 20 mg grain pellet food reward (TestDiet). Nose pokes on the inactive port were logged but had no consequence. Acquisition was conducted in a dual‐choice fixed‐ratio 1 (FR1) reinforcement schedule for 72 h. During acquisition, ad libitum chow was present in the home cage. PRL was conducted for 4 days following acquisition, wherein reward likelihood was 80% or 20% on each nose poke port and switched after 80 successful trials. During reversal learning, no chow was present in the home cage. Closed‐economy PR was conducted for 24 h, wherein subsequent trials required an increasing number of nose pokes on the active port to yield a grain pellet reward. After 30 min of no pellet earned, the FR contingency resets to 1.

### 2.3 Experimental Design and Statistical Analysis

Data from FED3 were written to a secure digital (SD) card on each behavioral device, and those data were processed and plotted with custom Python scripts. Comparisons between two samples were conducted with Mann–Whitney *U* tests (Python scipy.stats). Comparisons in response efficiency over time were analyzed using two‐way ANOVA with group and pellet as fixed factors, including their interaction (group × pellet). Significant main effects were followed by Tukey's HSD post hoc tests to identify specific differences. For permutation analyses, to assess the statistical significance of group differences, we fitted the RL model parameters on data with permuted control and wgl classifications and computed *p* values by summing the tail of their distribution beyond the value of the RL model parameters fit on the actual data [21].

### 2.4 Modeling

We used a modified Rescorla–Wagner RL model with choice kernel to analyze rodent decision‐making behavior across experimental groups [17, 22]. This model incorporates four key parameters: a learning rate (alpha) which updates based on reward prediction errors, an inverse temperature (beta) that scales the impact of rewards on choice behavior, a choice kernel learning rate (alpha_c) that captures the tendency to repeat previously selected actions independent of rewards, and a choice kernel inverse temperature (beta_c) that determines the strength of this perseveration effect. Initial action values were set based on the acquisition choice from each individual. Parameters were fitted by minimizing negative log‐likelihood across all subjects within each sex and experimental group simultaneously, allowing for group‐level inference while maintaining individual variability in choice sequences.

The foundation of our model is the classical Rescorla–Wagner learning rule [17], which updates option values based on prediction errors:

$$Q_{t+1}^{k}=Q_{t}^{k}+\alpha \left(r_{t}-Q_{t}^{k}\right)$$

Our preliminary data indicated that the model fits better when incorporating choice kernel parameters [23] that capture the tendency to repeat previous actions independent of rewards:

$$CK_{t+1}^{k}=CK_{t}^{k}+\alpha_{c}\left(c_{t}^{k}-CK_{t}^{k}\right)$$

The final model combines Rescorla–Wagner learning with choice kernel effects in the softmax probability function:

$$p_{t}^{k}=\frac{\exp\left(\beta Q_{t}^{k}+\beta_{c}CK_{t}^{k}\right)}{\sum_{i=1}^{k}\exp\left(\beta Q_{t}^{i}+\beta_{c}CK_{t}^{i}\right)}$$

## 3 Results

### 3.1 Diet‐Induced Obesity and Subsequent Weight Loss Model

**FIGURE 1:**
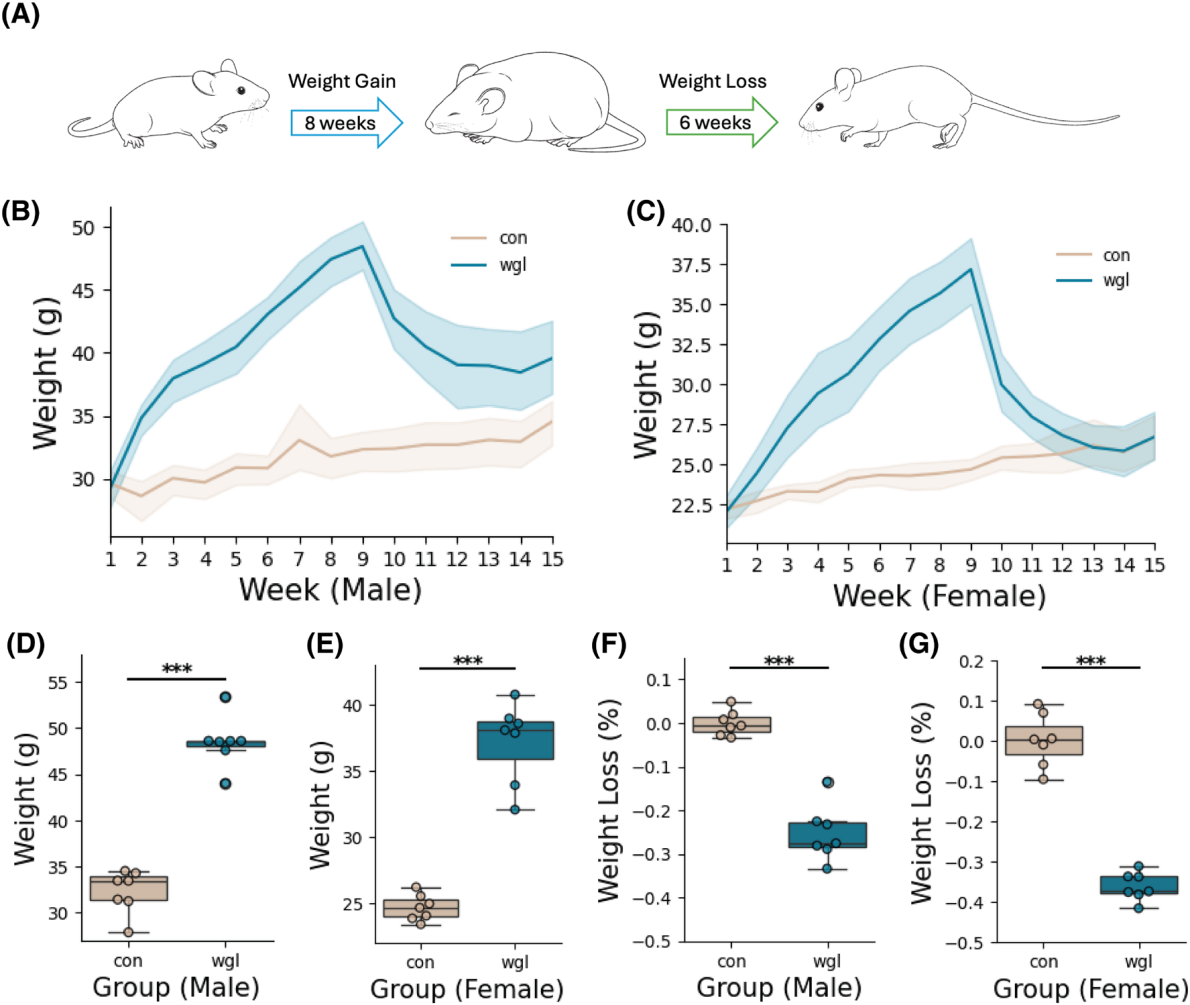
Weight loss after diet‐induced obesity model in mice. (A) Illustration of diet‐induced obesity model and subsequent weight loss paradigm. Control (con) group was provided regular laboratory chow food. Weight gain loss (wgl) group was provided high‐fat diet during the weight gain phase and regular chow during the weight loss phase. (B, C) Weight curves over 14 weeks by groups. (D, E) Weight at Week 9. For both males and females, the wgl group weighs significantly more than controls. (F, G) Normalized weight loss percentage (−avg of control weight). For both males and females, the wgl group weighs significantly more than controls. ***p < 0.001. [Color figure can be viewed at wileyonlinelibrary.com]

Male and female C57Bl6J mice (*n* = 7 mice per sex per group) were either provided with ad libitum 60% HFD or ad libitum chow for 8 weeks, followed by 6 weeks of exposure to ad libitum chow, and weighed weekly, to establish weight gain/loss (wgl) or control (con) groups (Figure 1A–C). Weight curves over the 14‐week period demonstrate that wgl mice rapidly gained weight during the high‐fat feeding phase, peaking around Weeks 8 and 9, followed by weight loss during the 6‐week regular chow phase (Figure 1B–G). Mice in the wgl group exhibited significant weight gain during the 8‐week HFD phase compared to control (con) mice fed regular laboratory chow, with final weights at Week 9 significantly higher in both males (*p* < 0.001, Figure 1D) and females (*p* < 0.001, Figure 1E). Normalized weight loss percentage at Week 9 was significantly different between groups for both males (*p* < 0.001, Figure 1F) and females (*p* < 0.001, Figure 1G).

### 3.2 PRL

#### 3.2.1 Acquisition

**FIGURE 2:**
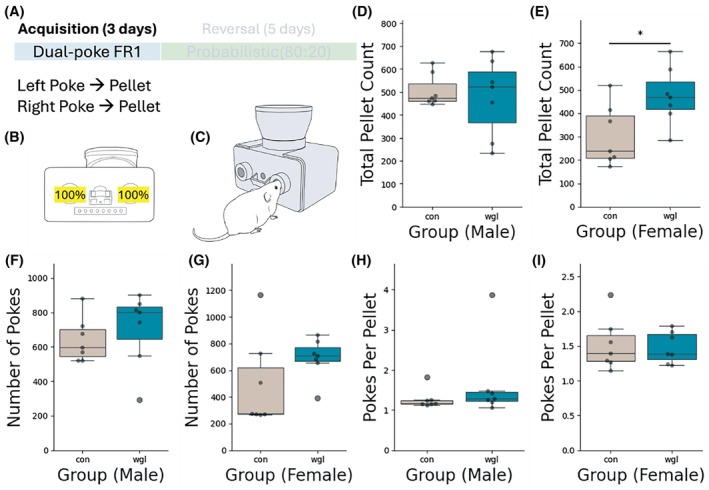
Acquisition of operant responding in a dual‐poke fixed‐ratio (FR1) task. (A) Experimental design showing the acquisition phase (3 days) with dual‐poke FR1 schedule where both left and right pokes resulted in pellet delivery. (B) Schematic of the dual‐poke FR1 task with 100% reinforcement for both left and right nose pokes during the acquisition phase on the FED3 device. (C) Illustration of a mouse performing the nose poke task on FED3. (D,E) Total pellet count during acquisition for male and female mice in control and wgl groups. A significant difference (*p < 0.05) was observed between groups in females, with wgl females obtaining more pellets than controls. (F, G) Number of nose pokes performed during acquisition by male and female mice in control and wgl groups. (H, I) Response efficiency measured as pokes per pellet for male and female mice in control and wgl groups. Data presented as box plots, and statistical analyses were performed using the Mann‐Whitney *U* tests for all comparisons. [Color figure can be viewed at wileyonlinelibrary.com]

Following weight loss, mice were exposed to 3 days of a FR1 reinforcement schedule wherein a nose poke on either the left or right port of a two‐choice home cage nose poke task yielded a 20 mg grain pellet reward (Figure 2A–C). Ad libitum chow was also present for the duration of the FR1 acquisition. Male wgl mice showed no difference in pellet acquisition compared to controls (Figure 2D), while female wgl mice earned significantly more total pellets than con female mice (Figure 2E). There was no significant difference between groups when comparing the number of nose pokes (Figure 2F,G) or pokes per pellet (Figure 2H,I) for either sex. This demonstrates that wgl and con mice of both sexes successfully learned to nose poke for grain pellet rewards, with no overt differences in efficiency.

#### 3.2.2 Reversal

**FIGURE 3:**
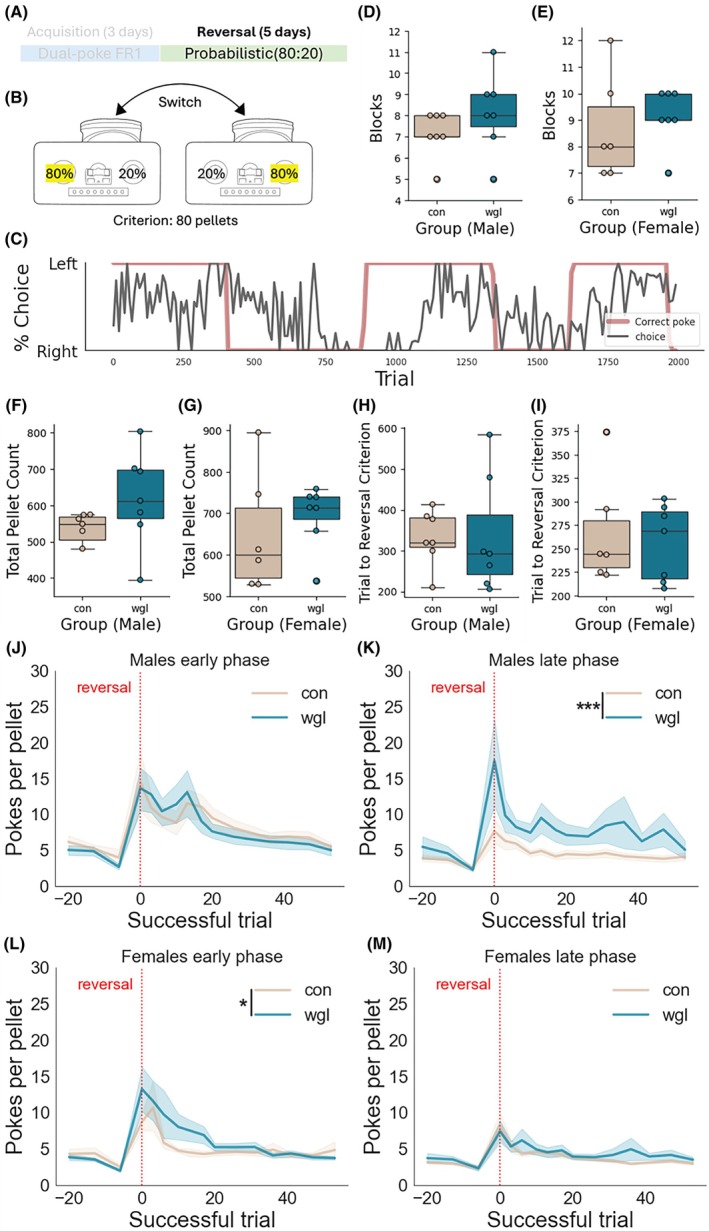
Performance during probabilistic reversal learning in a dual‐nose poke FED3 device. (A) Experimental design showing after acquisition, the reversal phase (5 days) with a probabilistic (80:20) reinforcement contingency. (B) Schematic of the reversal learning paradigm illustrating the contingency switch, where the previously less‐rewarded side (20%) becomes the high‐rewarded side (80%) and vice versa. Criterion for reversal was set at 80 pellets. (C) Representative choice trace (Partial) from a single mouse showing the percentage between left and right pokes across trials, with pink line indicating the correct poke option through the session. (D, E) Number of blocks completed by male and female mice in the control and wgl groups. (F, G) Total pellet count during reversal learning for male and female mice in control and wgl mice. (H, I) Trials to reversal criterion (80 pellets) for male and female mice in control and wgl groups. Mann‐Whitney *U* test showed no significant differences between groups in either sex (panels D–I). Data were divided into early and late temporal phases. Response efficiency analysis by pokes per pellet ratio aligned to reversal point (red dotted line) for males during (J) early and (K) late phases and females during (L) early and (M) late phases of reversal learning. Repeated measure ANOVA revealed significant group differences in males during the late phase (***p < 0.001) and females during the early phase (*p < 0.05), with wgl mice showing higher response rates (less efficient) compared to control mice. Data presented as either box plots with median or line graphs with mean ± standard error. [Color figure can be viewed at wileyonlinelibrary.com]

**FIGURE 4:**
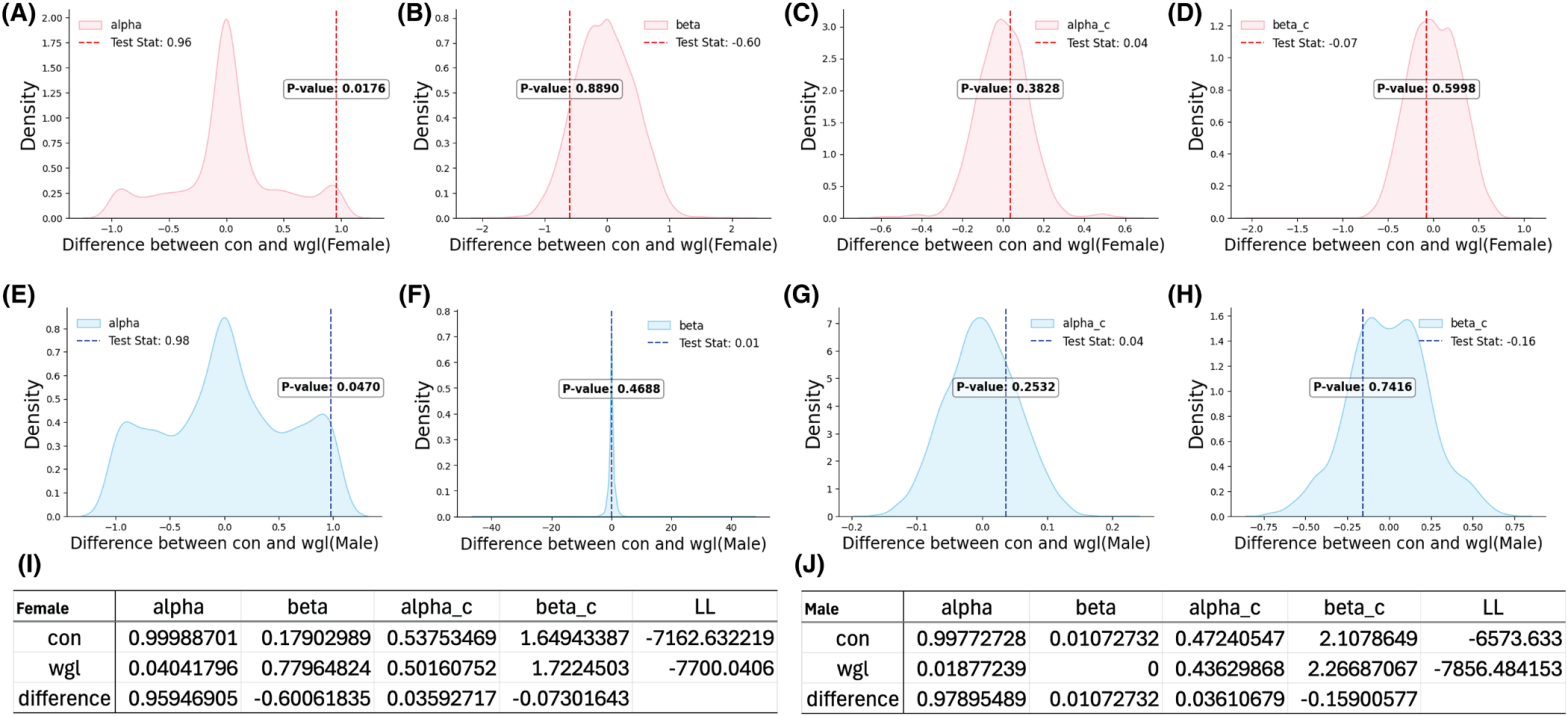
Reinforcement learning model analysis of probabilistic reversal learning performance. Permutation test results (5000 permutations) showing distribution of parameter differences between randomly assigned groups for female mice: (A) learning rate *α* (Test Stat: 0.96, *p* = 0.0176), (B) inverse temperature *β* (Test Stat: −0.60, *p* = 0.8890), (C) choice kernel learning rate *α*_c (Test Stat: 0.04, *p* = 0.3828), and (D) choice kernel inverse temperature *β*_c (Test Stat: −0.07, *p* = 0.5998). Red vertical lines indicate actual con‐wgl difference values. Permutation test results (5000 permutations) showing distribution of parameter differences between randomly assigned groups for male mice: (E) *α* (Test Stat: 0.98, *p* = 0.0470), (F) *β* (Test Stat: 0.01, *p* = 0.4688), (G) *α*_c (Test Stat: 0.04, *p* = 0.2532), and (H) *β*_c (Test Stat: −0.16, *p* = 0.7416). Red vertical lines indicate actual con‐wgl difference values. Model parameter values and log‐likelihood (LL) for (I) female and (J) male mice. In females, control mice showed *α* = 0.999, *β* = 0.179, *α*_c = 0.537, and *β*_c = 1.649, while wgl mice showed *α* = 0.040, *β* = 0.780, *α*_c = 0.502, and *β*_c = 1.722. In males, control mice showed *α* = 0.998, *β* = 0.011, *α*_c = 0.472, and *β*_c = 2.108, while wgl mice showed *α* = 0.019, *β* = 0, *α*_c = 0.436, and *β*_c = 2.267. Data presented as density distributions with test statistics indicated by vertical lines. [Color figure can be viewed at wileyonlinelibrary.com]

After acquisition, we asked whether wgl mice would perform differently in a task of cognitive flexibility. To do this, we exposed wgl and con mice to the PRL task for 4 days following FR1 acquisition (Figure 3A). The PRL task was conducted in a closed‐economy setting wherein no chow was present in the home cage, and mice acquired all caloric content from the task. In the PRL paradigm, a left‐hand nose poke choice is rewarded 80% of the time, and a right‐hand poke 20% of the time. Every 80 rewarded trials, the contingency reverses (Figure 3B,C). Wgl and con groups completed similar numbers of blocks (Figure 3D,E) and earned similar numbers of pellets (Figure 3F,G). There was no significant difference in the number of trials needed to reach the reversal criterion between groups (Figure 3H,I). Data were divided into two learning phases, early (first 45 h) and late (second 45 h). However, a response efficiency analysis revealed male wgl mice exhibited significantly higher pokes per pellet during the late phase of reversal learning (*p* < 0.001, Figure 3K), while female wgl mice showed impaired efficiency during the early phase (*p* < 0.05, Figure 3L). We conclude that wgl mice exhibit reduced adaptive learning rates relative to con. To further test this, we applied an RL model analysis and compared computational parameters alpha, beta, alpha_c, and beta_c between groups within each sex (Figure 4A,B). Female wgl mice showed dramatically reduced learning rates (*α* = 0.004) compared to controls (*α* = 0.999, Figure 4C,K). Similarly, male wgl mice demonstrated markedly lower learning rates (*α* = 0.019) compared to controls (*α* = 0.998, Figure 4G,L). There was no significant difference in beta, alpha_c, or beta_c parameters (Figure 4D–F,H–L). These differences in learning rate suggest that previously obese mice exhibit fundamental alterations in how they process and respond to recent choice outcomes during probabilistic RL, despite having returned to normal weight.

#### 3.2.3 PR Task

**FIGURE 5:**
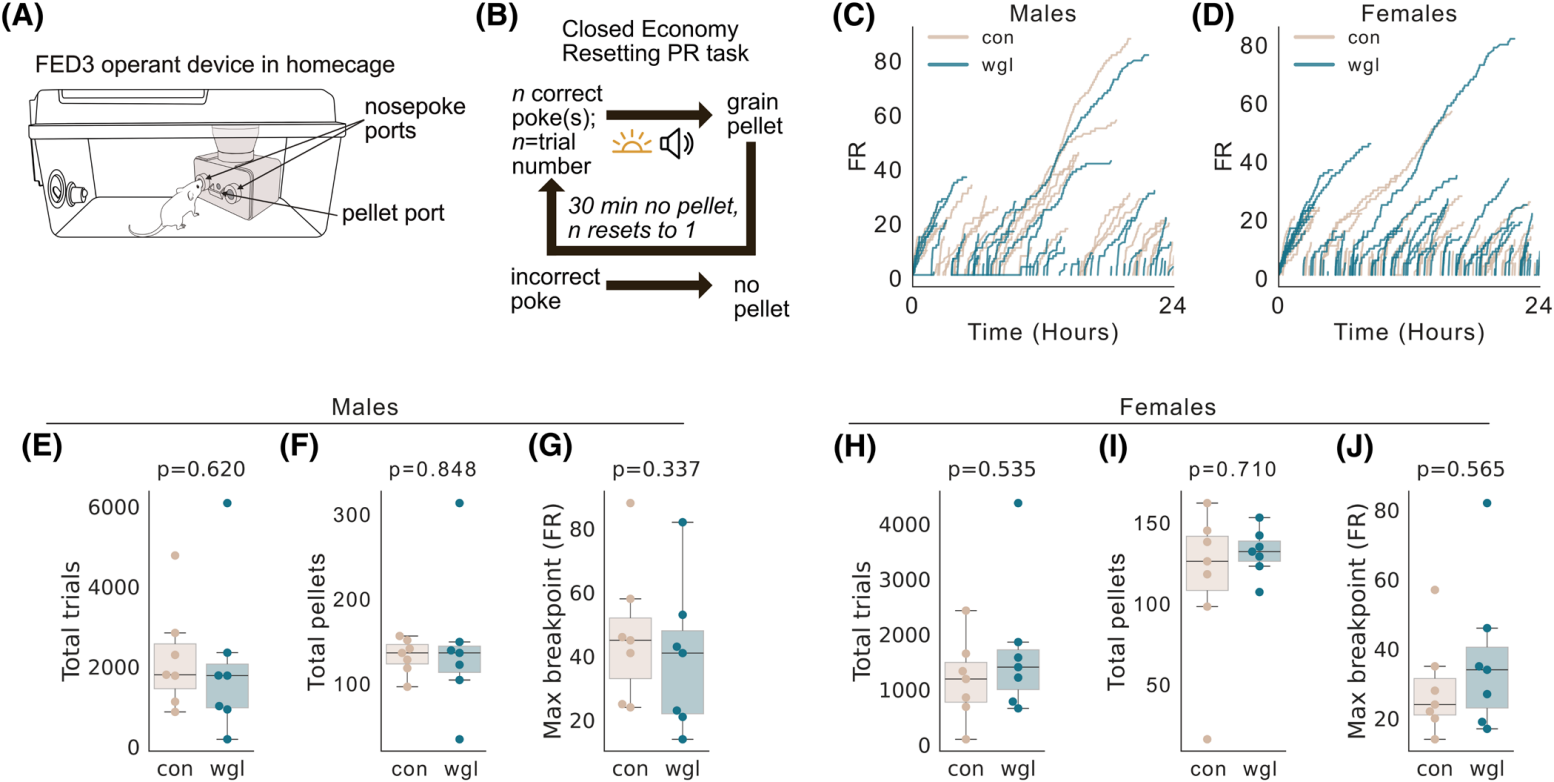
Weight loss after obesity does not change effort output for food in a closed‐economy task. (A) Schematic depicting the FED3 operant device in the home cage. Grain pellets were the only source of food during the task. (B) Mice were tested on a resetting PR task wherein a successful nose poke was rewarded with a 20 mg grain pellet. Subsequent trials required an increased number of pokes. After 30 min without a poke, the requirement resets to 1. (C, D) All trial runs for male and female mice throughout the experiment. Required FR increases until a 30‐min break institutes a reset. No difference in overall trials completed between groups for (E) males or (H) females (*p* = 0.620 and 0.535, respectively). No difference in overall pellets earned between groups for (F) males or (I) females (*p* = 0.848 and 0.710, respectively). No difference in breakpoint between groups for (G) males or (J) females (*p* = 0.337 and 0.565, respectively). In panels E–J, the Mann‐Whitney *U* test is performed. [Color figure can be viewed at wileyonlinelibrary.com]

Obesity is associated with decreased motivation [24, 25, 26]. To ask whether wgl mice exhibited obesity‐like decreased motivation for grain pellets, which may have skewed their performance in a reversal learning paradigm, we exposed mice to a closed‐economy PR nose poking task for grain pellets for 24 h. A nose poke on the correct side yielded a 20 mg grain pellet reward, and subsequent trials (*n*) required an increasing number of pokes (*n* + 1) to receive a grain pellet. After 30 min of no pellet earned, the task reset to a requirement of FR = 1. All calories were obtained from the feeding device, as no chow was present in the cage. We observed that for both males and females, there was no significant difference in trials performed (Figure 5E,H), pellets earned (Figure 5F,I), or breakpoint (Figure 5G,J) between con and wgl mice. We conclude that motivation to consume grain pellets is not decreased in the weight loss state, and that wgl mice exhibit intact baseline motivations toward homeostatic needs.

## 4 Discussion

In this study, we aimed to investigate whether diet‐induced obesity induced persistent alterations in cognitive flexibility, which plays a critical role in weight loss maintenance in people [27, 28]. By integrating a reversal learning behavioral task and an RL model, our results demonstrate that a history of obesity induces persistent deficits in reward‐guided decision‐making in a sex‐dependent manner, even after body weight recovers. Our results identify a potential behavioral basis for vulnerability to relapse after successful weight loss.

### 4.1 Persistence of Cognitive Inflexibility After Weight Loss

We used a mouse model of post‐obesity weight loss that transitions from chronic HFD exposure to a weight loss phase with chow diet. We found that both female and male mice showed marked reductions in performance efficiency on PRL tasks following weight loss, demonstrating that weight loss after obesity induces persistent, sex‐specific deficits in cognitive flexibility (Figure 3K,L).

Clinical observations of high weight regain rates among people who have lost weight after obesity suggest cognitive inflexibility—rooted in altered RL strategies—is present in people with obesity as well and may contribute to the challenge of maintaining weight loss [28, 29]. In our RL model, both male and female wgl mice showed reductions in learning rate (alpha) compared to controls, indicating a diminished capacity to update value representations in response to prediction errors, while other parameters (i.e., beta) maintained intact. This deficit occurred despite recovered body weight, suggesting that prior obesity induces long‐lasting maladaptations in reward processing. In people, impaired cognitive flexibility in the weight loss state may prevent timely adaptation to changing environments (e.g., adjusting to satiety cues). These dynamics also mirror addiction models, where cognitive rigidity and altered reward valuation perpetuate relapse [30].

### 4.2 Weight Loss After Obesity Does Not Impact Performance in FR or Closed‐Economy PR Tasks

In addition to the PRL task, we conducted a FR operant task during acquisition (Figure 2), as well as a closed‐economy PR task (Figure 5). We did not observe overwhelming differences between the wgl and con groups in outcomes from FR and closed‐economy PR tasks. This differs from reports of rodent behavior in obese states that show diminished responding on similar fixed or progressive reinforcement schedules [31, 32] and suggests such operant learning mechanisms are distinct across obesity and weight loss states. Comparable performance between the wgl and con groups in acquisition on a FR schedule supports that initial associative learning mechanisms are not impaired in the weight loss state, supporting previous studies [33]. No observed difference between groups in breakpoint observed in the closed‐economy PR task supports that con and wgl mice did not display significant differences in motivation to work for grain pellets. This is counter to what we have observed in previous studies, where mice in the weight loss state worked harder on a similar PR task for sucrose pellets [33]. Taken together, this suggests that while enhancements in motivation to work for palatable food, such as sucrose, occur in a weight loss state [33], motivation to work for grain pellets in a closed‐economy setting is unchanged between weight loss and control. And while we observe decreases in learning rate between wgl and con mice (Figures 3 and 4), data from the FR and PR tasks support that cognitive function is not impaired in the weight loss state.

Interestingly, data from the FR task (Figure 2), the probabilistic reversal task (Figure 3), and the closed‐economy task (Figure 5) in which all food is acquired through interaction with the device did not reveal differences in the total amount of grain pellets earned, with the exception of female wgl mice earning significantly more pellets during FR acquisition than female con mice (Figure 2E). This suggests broadly that there was no difference between wgl and con mice in the consumption of grain pellets, despite reduced efficiency in the probabilistic reversal task. A decrease in efficiency indicates that mice had to perform more actions to obtain the same amount of food—not that they consumed less. The fact that total intake remained unchanged suggests that wgl obese mice were motivated to meet their caloric needs but engaged in a more effortful or less optimized behavioral strategy to do so. This is consistent with impaired cognitive flexibility—rather than adapting their responses quickly when contingencies changed, wgl mice continued to rely on outdated or inefficient strategies, requiring more trial‐and‐error to obtain rewards. Thus, the reduction in efficiency reflects a cognitive processing deficit (e.g., slower value updating), not diminished appetite or motivation. This dissociation supports a model of obesity wherein obesity induces a lasting change in decision‐making strategies that persists after weight loss and may hinder goal‐directed behavior in complex or uncertain environments, even when caloric drive is intact.

### 4.3 Sex‐Specific Effects of Weight Loss on Cognitive Flexibility

Our results demonstrate that males exhibit deficits in behavioral efficiency during the late phase of PRL (Figure 3K), while females show early‐phase inefficiencies but recover by the late phase (Figure 3L). This is aligned with sex differences in the Iowa gambling and Soochow gambling tasks: females tend to focus on recent outcomes and gain/loss frequency, while males attend to extreme outcomes and long‐term patterns [34]. Thus, female mice might incorporate recent information updates to adjust their behaviors more rapidly than males. Our results extend sex‐specific decision‐making frameworks to weight loss after obesity, with a highlight on how females may compensate for early deficits through altering strategies, including integrating immediate recent information, while males struggle to disengage from prior strategies.

### 4.4 Limitations

There are limitations to this study. First, various interventions for weight loss might lead to different cognitive effects. For instance, pharmacological interventions such as GLP1‐R agonists (semaglutide/Ozempic) may differentially influence cognitive processes after weight loss in diet‐induced obese mice, which we did not test here. Additionally, uncovering neural mechanisms will be critical to reveal the biological foundation of cognitive inflexibility in the weight loss state and could provide a specific target for pharmacological interventions to achieve an efficient on‐site drug delivery. For example, the prelimbic cortex to nucleus accumbens pathway has been implicated in reversal learning tasks in rats [35], and accumbal activity and prefrontal cortical activity are known to be disrupted in rodents and people with obesity [18, 36, 37, 38, 39, 40, 41, 42, 43]. Human studies report inconsistent effects of obesity on PL‐NAc pathway activity. Some have found increased PL activity in individuals with obesity compared to lean controls [40, 41, 43, 44], while others report decreased PL activity [45, 46, 47]. In rodent models, obesity disrupts NAc synaptic plasticity [36, 48, 49] and disinhibits frontal cortical projection neurons [50, 51], both consistent with altered PL‐NAc signaling. Two in vivo rodent studies have directly examined obesity‐induced changes in striatal dynamics: one found that HFD‐induced obesity reduced PL‐NAc coherence [52], while the other showed diminished movement‐related striatal activity in obese mice relative to lean controls [53]. Together, these findings suggest that obesity disrupts corticostriatal function—particularly within the PL‐NAc circuit—but it remains unknown whether such alterations persist following weight loss or abstinence from obesogenic diets. A study investigating the role of the PL‐NAc brain circuit in cognitive flexibility tasks in obesity and weight loss states may reveal a neural substrate underlying our findings here.

Sex hormones regulate learning and memory [54, 55], suggesting the estrous cycle could influence female performance on reversal learning tasks. A limitation of our study is that we did not monitor the estrous cycle during the testing period. However, prior work offers mixed evidence regarding its impact: while ovariectomy altered choice behavior in an effort discounting task, intact females showed no variation in decision‐making across the estrous cycle [56]. Similarly, risky decision‐making did not significantly differ across the estrous cycle in female rats or across the menstrual cycle in human women [57, 58]. Conversely, more recent findings indicate that choice impulsivity may fluctuate across the estrous cycle in rats, though there was not a direct relationship between sex hormone levels and reward timing processes like delay discounting [59].

Finally, here we did not test whether reduced learning rate (*α*) predicts or correlates with weight regain. This remains a key translational gap, as impaired value updating may hinder adaptation to real‐world challenges (e.g., holiday eating and stress), increasing relapse risk. Longitudinal studies linking cognitive flexibility measures to post‐intervention weight trajectories—for instance, with re‐exposure to an obesogenic diet after initial weight loss—could identify computational markers of vulnerability and determine whether improving flexibility (e.g., through cognitive training) supports long‐term weight maintenance.

## Conflicts of Interest

The authors declare no conflicts of interest.

## Data Availability

The code and data that support the findings of this study are openly available in the Open Science Framework at https://osf.io/ma9bv.
